# Network of miR396-mRNA in Tissue Differentiation in Moso Bamboo (*Phyllostachys edulis*)

**DOI:** 10.3390/plants12051103

**Published:** 2023-03-01

**Authors:** Ying Li, Naresh Vasupalli, Ou Cai, Xiaofang Lin, Hongyu Wu

**Affiliations:** 1 National State Forestry and Grassland Administration Key Open Laboratory on the Science and Technology of Bamboo and Rattan, Institute of Gene Science and Industrialization for Bamboo and Rattan Resources, International Centre for Bamboo and Rattan, Beijing 100102, China; 2 Bamboo Industry Institute, Zhejiang Agriculture and Forestry University, Hangzhou 311300, China; 3 Co-Innovation Center for Sustainable Forestry in Southern China/Bamboo Research Institute, Nanjing Forestry University, Nanjing 210037, China

**Keywords:** miR396, tissue differentiation, network, transcription factor, bamboo shoot, seedling

## Abstract

MiR396 plays an essential role in various developmental processes. However, the miR396-mRNA molecular network in bamboo vascular tissue differentiation during primary thickening has not been elucidated. Here, we revealed that three of the five members from the miR396 family were overexpressed in the underground thickening shoots collected from Moso bamboo. Furthermore, the predicted target genes were up/down-regulated in the early (S2), middle (S3) and late (S4) developmental samples. Mechanistically, we found that several of the genes encoding protein kinases (PKs), growth-regulating factors (GRF), transcription factors (TFs), and transcription regulators (TRs) were the potential targets of miR396 members. Moreover, we identified QLQ (Gln, Leu, Gln) and WRC (Trp, Arg, Cys) d omains in five *PeGRF* homologs and a Lipase_3 domain and a K_trans domain in another two potential targets, where the cleavage targets were identified via degradome sequencing (*p* < 0.05). The sequence alignment indicated many mutations in the precursor sequence of miR396d between Moso bamboo and rice. Our dual-luciferase assay revealed that ped-miR396d-5p binds to a *PeGRF6* homolog. Thus, the miR396-GRF module was associated with Moso bamboo shoot development. Fluorescence in situ hybridization localized miR396 in the vascular tissues of the leaves, stems, and roots of pot Moso bamboo seedlings at the age of two months. Collectively, these experiments revealed that miR396 functions as a regulator of vascular tissue differentiation in Moso bamboo. Additionally, we propose that miR396 members are targets for bamboo improvement and breeding.

## 1. Introduction

Bamboo is a member of the subfamily Bambusoideae of Poaceae grass and is characterized by a complex and well-developed underground system, where buds germinate at the nodes of rhizomes in autumn, expand gradually until the spring of the following year and ultimately develop into mature aboveground shoots [1]. China has the world’s most abundant bamboo resources and contains the highest number of Bamboo species and forest areas [2]. Bamboo forests contribute to multiple ecological and economic benefits in the form of soil erosion control, water storage, food, and paper, which are of great significance to economic development, rural revitalization and ecological civilization construction [3]. Furthermore, bamboo is unique in its capacity for carbon sequestration. The average annual carbon sequestration amount of bamboo forests is 1.5 times that of Chinese fir plantations and 1.33 times that of tropical rainforests [4]. In addition, Bamboo forests act as natural reservoirs because of their whip-root system [5]. Through interception, absorption and protection of the forest canopy, understory vegetation and root-soil, the whip-root system of bamboo forests helps prevent water precipitation. Therefore, it improves the groundwater levels and the soil water storage capacity [6]. Moreover, bamboo shoots are edible vegetables with high nutritional value [7], rich in flavonoids, polysaccharides and other bioactive substances [8]. In addition to bamboo shoots, various understory products have become common food materials in daily life [9].

*Phyllostachys edulis* (Moso bamboo) is China’s most essential bamboo forest species, accounting for 72.96% of the total bamboo forest area [10]. Interestingly, its buds exhibit primary thickening. Moreover, the volume of its mature shoots, a delicious forestry food product, is generally dozens of times larger than its buds. The mature shoots sprout out of the ground, then quickly elongate and grow into a culm with constant node numbers and coarseness. To a large extent, primary thickening determines the shoot taste and the nutrient composition, wood properties, and the aboveground culms’ morphological features. Previous studies have shown that the primary thickening of bamboo depends on the maintenance and organogenesis of the shoot apical meristem. Primary thickening is accompanied by diverse physiological and biochemical changes, such as changes in carbohydrate metabolism [11], enzyme activities [12], and hormone concentrations [13]. Clearly, primary thickening is a complicated developmental process affected by endogenous and environmental factors.

In the last few years, with the rapid development of high-throughput sequencing and multi-omics analysis, it has become evident that a complex network regulates primary thickening growth. Recently, in the underground shoots of Moso bamboo and its natural mutants, Wei et al. [14,15] identified that the genes involved in cell wall configuration, plant hormone signal transduction, and other related processes are highly enriched. These results indicated an essential role of tissue differentiation in the early stage of primary thickening. Further, Li et al. [16] identified the genes and miRNAs in the underground shoots of Moso bamboo and its wall-thickness variant (*P. edulis* f. ‘pachyloen’) at five different developmental stages via a comprehensive multi-genome analysis. In addition, they also screened miRNA-mRNA pairs regulating shoot development and found cytokinin levels vary dramatically over five stages. Although hundreds of miRNA-mRNA pairs were identified and quantified in the primary thickening shoots, except for miR166, the miRNA-mRNA networks, expression patterns, and tissue localization are still unclear. Therefore, it is critical to gain new insights into the miRNA-mRNA molecular networks of the biological pathways and elucidate the species specificity in bamboo.

Growth-regulating factors (GRFs) in the transcription factor family are critical in promoting various plant developmental processes. Further, the expressions of most GRFs were negatively regulated at the post-transcriptional level by miR396 by directly binding the mRNA transcripts, followed by cleaving the mRNA and inhibiting the translation [17]. The conserved miR396-GRF regulatory module is critical for coordinating plant growth with the endogenous and environmental factors [18]. In *Arabidopsis thaliana*, the ectopic expression of the miR396-GRF regulatory module as a developmental regulator affects multiple important biological processes, including embryogenic responses [19], cell proliferation in leaf primordia [20], leaf development [21], and root cell reprogramming [22,23,24]. In rice, the miR396-GRF regulatory module also impacts rice architecture [25] and grain size and yield [12,26]. Recently, Kim et al. [27] indicated that the decrease in rice (Nipponbare) leaf blade size was related to augmented CO_2_ and the miR396-GRF module. This module also functions in other species, including poplar (*Populus trichocarpa*) and switchgrass (*Panicum virgatum*), affecting the organ type during flower development, the biomass, and the feedstock quality. Our previous report showed that miR396 and several putative GRF genes were differentially expressed in the underground primary thickening shoots of wild-type (WT) Moso bamboo as well as its thick-wall (TW) variant (*P. edulis* ’Pachyloen’) [16]. These reports indicate that the miR396-GRF module is essential for bamboo development. However, the miR396-mRNA network in Moso bamboo plants is still unclear, and the role of miR396 in the tissue differentiation of bamboo plants remains elusive.

Therefore, we hypothesized that (i) the miR396 family members play an essential role in the primary thickening of underground shoots because of their high expressions throughout all five stages; (ii) the target genes of Moso bamboo miR396 might be involved in multiple regulatory pathways to optimize tissue development and differentiation during the underground thickening of the shoots; and (iii) the miR396s function might be asynchronous with other miRNA families to regulate bamboo shoot thickening together. Since the period of bamboo shoot thickening is long, at least through three different seasons, only a multi-factor, multi-level, multi-period complex regulatory network could ensure the continuity and accuracy of this process. To test these hypotheses, we identified and quantified the miR396 members of the underground WT and TW of Moso bamboo shoots at the five developmental stages, predicted the target genes, and elucidated the miR396-mRNA network based on comprehensive multi-omics analyses. We also confirmed the interaction between miR396s and a putative *PeGRF* gene. Finally, we verified the localization of miR396s in the vascular tissues of two-month-old bamboo seedlings and assessed their potential contribution to morphological differences in Moso bamboo. Our findings will help us better understand the function of miR396 in tissue differentiation and identify candidate genes to improve bamboo biomass and wood properties.

## 2. Results

### 2.1. A Total of 1128 Predicted Target Genes of Five miR396 Members

**Figure 1:**
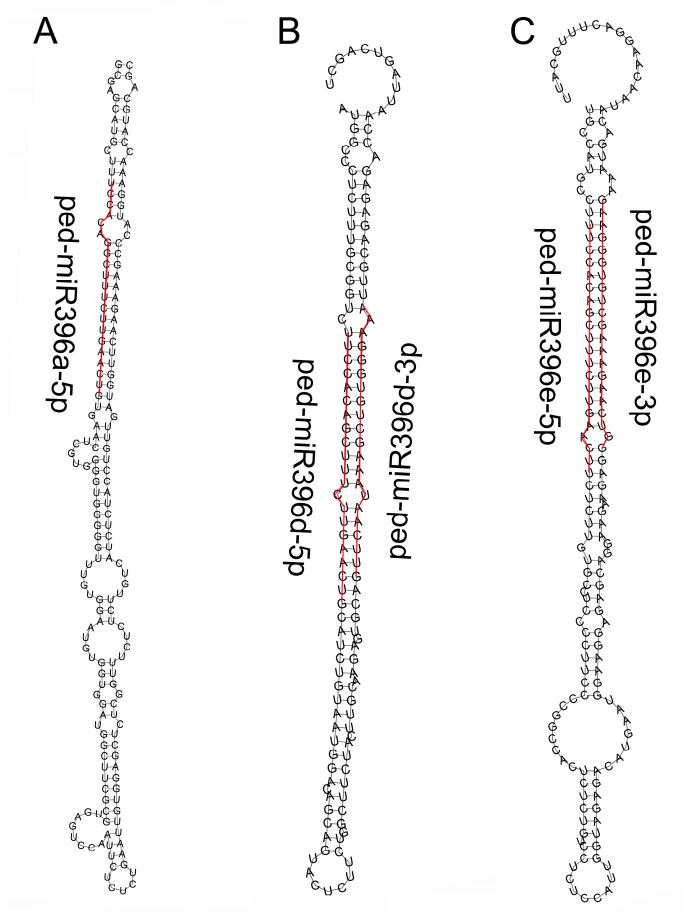
Secondary structures of the five miR396 members predicted using online software. The mature miR396 sequences are delineated by the red lines. (**A**–**C**) Predicted secondary structures of ped-miR396a-5p, ped-miR396d-5p, ped-miR396d-3p, ped-miR396e-5p, and ped-miR396e-3p.

In the current study, we used the five developmental stages, i.e., germination, early, middle, late and mature stages (WTS1/TWS1-WTS5/TWS5) transcriptome and miRNA data from our previous publication [16] for identifying the miR396 members and their predicted target genes. A total of five 21 nt long miR396 members (Table S1) were identified from the miRNA data. The average GC content of miR396 members was 43.81%. The minimum free energy of secondary structures ranged from −71.20 to −62.90 kcal/mol for ped-MIR396a and ped-MIR396d, respectively (Figure 1). In addition, 5′ uridine was found at the first base of three of the five (60.00%) miR396s.

Five miRNAs were predicted to have 1285 target genes (Table S2). Of these target genes, 44 encoded transcription factors (TFs) of 14 families. Among these TF genes, 20 (45.45%) encoded GRFs, 4 (9.09%) encoded homeobox-homeodomain-leucine zipper (HB-HD-ZIP), 4 (9.09%) encoded basic region/leucine zipper motif (bZIP), 3 (0.41%) encodedd MADS-MIKC, 67 genes encode protein kinases (PKs), and 7 encoded transcription regulators (TRs).

### 2.2. Three Up-Regulated miR396s in Bamboo Underground Shoots

All five miR396s had more than one transcript per million (TPM) reads in one or more WT or TW samples. Three (60.00%) miRNAs were found at all five developmental stages. Specifically, three miRNAs had more than 500 TPM reads at all developmental stages of the WT and TW samples (Table S1).

Furthermore, among the pairs composed of five miR396s and 1285 predicted targets, 536 involved ped-miR396e-5p, 443 involved ped-miR396d-5p, 284 involved ped-miR396a-5p, 191 involved ped-miR396e-3p, and 140 involved ped-miR396d-3p (Table S2).

**Figure 2:**
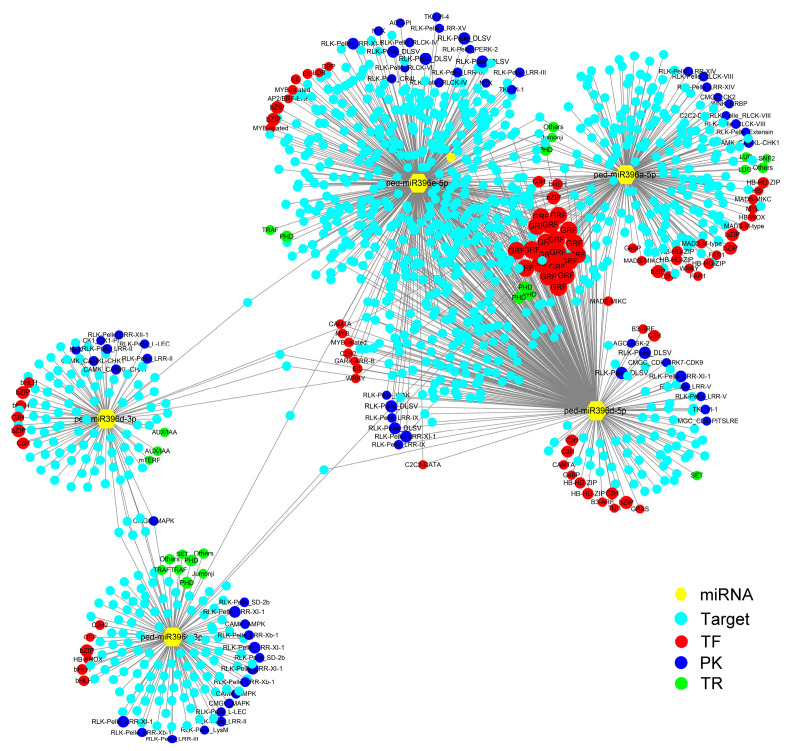
The regulatory network of the five miR396s and their predicted targets using Cytoscape. MiR396s and their target genes are indicated by yellow triangles and blue solid circles, respectively. Their targets encoding PKs, TFs, and TRs are indicated by deep blue, red, and green circles, respectively.

As shown in Figure 2, almost all three overexpressed miR396s targeted the GRF TF family besides other TF genes, such as HB-HD-ZIP, bZIP, and MYB. In addition, RLK-Pelle_DLSV participated in the most miR396-mRNA pairs (8).

### 2.3. Four Differentially Expressed miR396s in 13 Combination Pairs

Detailed analysis revealed that four (80.00%) miR396s, namely ped-miR396a-5p, ped-miR396d-5p/3p, and ped-miR396e-5p, were differentially expressed in 13 pairwise combinations with TPM > 1 in either of the two pairwise combination samples (FDR < 0.05 and |log2(FC)| ≥ 1) (Table S1). These four miR396s showed differential expressions in different pairwise combinations, but the pairwise combinations were all composed of samples collected at stages of S2–S4. For example, ped-miR396a-5p showed different expression in the WTS3_vs_WTS2, TWS4_vs_TWS3, and WTS3_vs_TWS3 pairs, while ped-miR396d-5p was differently expressed in TWS4_vs_TWS3, TWS3_vs_TWS2. Notably, three overexpressed miR396s were all differentially expressed in TWS3_vs_TWS2 and TWS4_vs_TWS3. After germination, during the S2–S4 stages, continuous tissue differentiation and thickening of bamboo shoots occurred.

**Figure 3:**
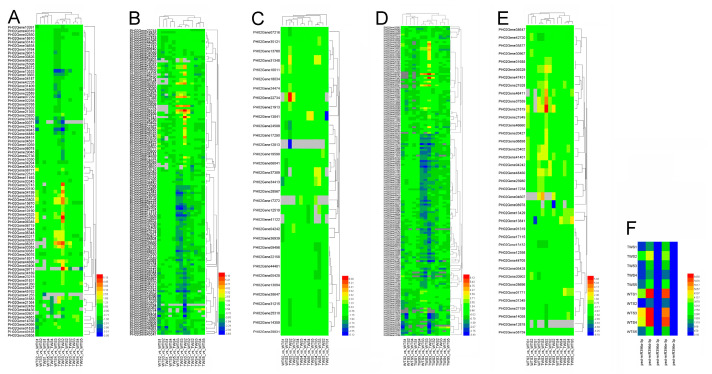
Expression patterns of five miR396s and their target genes that are differentially expressed in 13 combination pairs, including TWS5_vs_WTS5, TWS4_vs_WTS4, TWS3_vs_WTS3, TWS2_vs_WTS2, TWS1_vs_WTS1, TWS5_vs_TWS4, TWS4 _vs_TWS3, TWS3 _vs_TWS2, TWS2 _vs_TWS1, WTS5 _vs_WTS4, WTS4 _vs_WTS3, WTS3 _vs_WTS2, and WTS2 _vs_WTS1. (**A**–**E**) Target genes of five miR396s. (**F**) Five miR396s. Gray squares represent miRNAs and their downregulated genes with TPM ≤ 1 or FPKM ≤ 1.

Additionally, 85 target genes of the three DEmiR396s with TPM > 1 in either of the two pairwise combination samples (FDR < 0.05 and |log2(FC)| ≥ 1) were differentially expressed in at least one of the 13 comparison pairs. Furthermore, most of them were up- or down-regulated in the TWS4_vs_TWS3, TWS3_vs_TWS2, TWS2_vs_TWS1, WTS4_vs_WTS3, WTS3_vs_WTS2, WTS2_vs_WTS1, and WTS3 vs_TWS3 pairs (Figure 3). A total of 115 DEmiR396-DEmRNA interaction pairs were identified. Among these pairs, 57 were coherently expressed (Table S3).

### 2.4. Ten Confirmed MiRNA-mRNA Pairs by Degradome Sequencing

**Figure 4:**
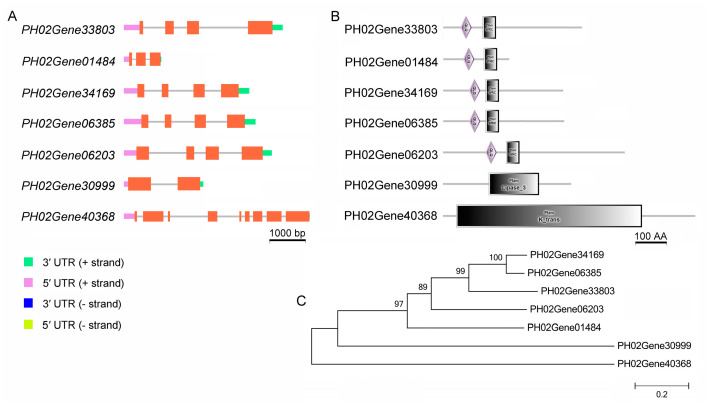
MicroR396s and their predicted targets in seedlings of Moso bamboo. (**A**) Shown are the sequences of microR396s, a protein kinase gene, and seven class III *HD-ZIP* genes; (**B**) Shown are the Class III *HD-ZIP* genes encoding proteins with a MEKHLA domain at the N-terminal, a HOX domain at the C-terminal, and a START domain; (**C**) Shown is a diagram representing the relationship among the seven target genes. Boxes symbolizes exons, lines represent introns, and the coding sequences represent three structural domains MEKHLA, HOX, and START. Shown below these domains are the sequences of the binding sites of miR396s to its seven targets. The bar equals 100 amino acid residues.

There were seven cleavage sites in the ten miRNA-mRNA pairs that involved the three miR396s and their seven targets from the WT and TW samples (Figure 4A, Table S4, (*p* < 0.05). Of the seven targets, five had a QLQ (Gln, Leu, Gln) domain and a WRC (Trp, Arg, Cys) domain and were clustered into one group (Figure 4B,C). The QLQ domain is at the N-terminus of the SWI2/SNF2 protein, which participates in protein-protein interactions; the WRC is present in GRF proteins, the transcription regulators in key developmental processes. Additionally, PH02Gene30999 had a Lipase_3 domain, which represents a domain with an alpha/beta hydrolase fold found in feruloyl esterase A and is involved in lipid metabolic processes (GO:0006629). Further, *PH02Gene40368* had a K_trans domain, which is involved in potassium ion transmembrane transport (GO:0071805).

Generally, miR396 members from the same family cleave the same target. For example, the putative rice PeGRF6 homolog encoded by *PH02Gene06203* was cleaved at the same site by ped-miR396a-5p from the WT samples, and ped-miR396d-3p from the TW samples (Table S4-1). Interestingly, although the same number of miR396-mRNA pairs was found in both the WT and TW samples (Table S4-2,4-3), they cleaved the same target gene differently. For example, *PH02Gene40368*, which carries a K_trans domain, was cleaved by ped-miR396e-5p from the WT but not from the TW, while *PH02Gene30999*, which carries a Lipase_3 domain, was cleaved by ped-miR396a-5p from the TW but not from the WT.

### 2.5. MiR396s Specifically Bind to PeGRF TF Genes and Are Localized in Vascular Tissues

**Figure 5:**
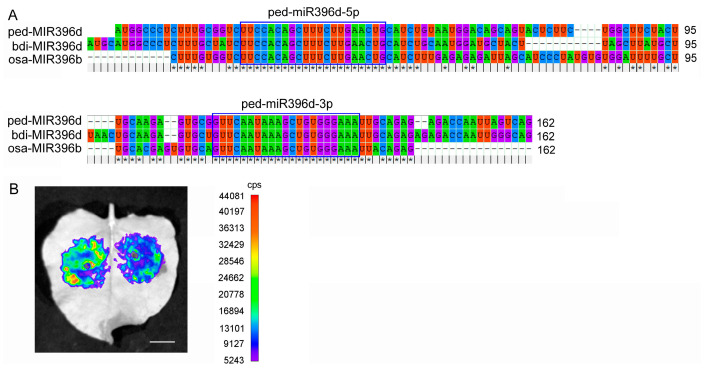
The correlation of three miR396s with a putative *PeGRF6* analyzed by dual-luciferase reporter and transient expression assays. (**A**) Alignment of precursor sequences of miR396d from rice, bamboo, and *Brachypodium distachyon*. Sequences of bdi-miR396d and osa-miR396d from *B. distachyon* and rice, respectively, were downloaded from the miRBase; (**B**) Fluorescence intensity of the same leaf injected with an empty vector (EV) + PeGRF6 at one side and with miR396 + PeGRF6 at the other side, indicating the binding of miR396 to *PeGRF6*. Bar = 1 cm.

As shown in Figure 5A, the mature sequences were conserved between the miR396d precursor sequences from rice, Moso bamboo, and *Brachypodium distachyon*. However, there were many mutations in the nucleotide sequences in the flanking sequence among the miR396d precursor sequences. Moreover, the transient expression assay indicated that ped-miR396d-5p binds to a putative *PeGRF6* homolog of rice (GenBank accession No:OQ026341). The fluorescence intensity was higher in the left-side leaf treated with empty vector + PeGRF6 than in the right-side leaf treated with miR396 + PeGRF6 under UV light in *N. benthamiana* (Figure 5B).

**Figure 6:**
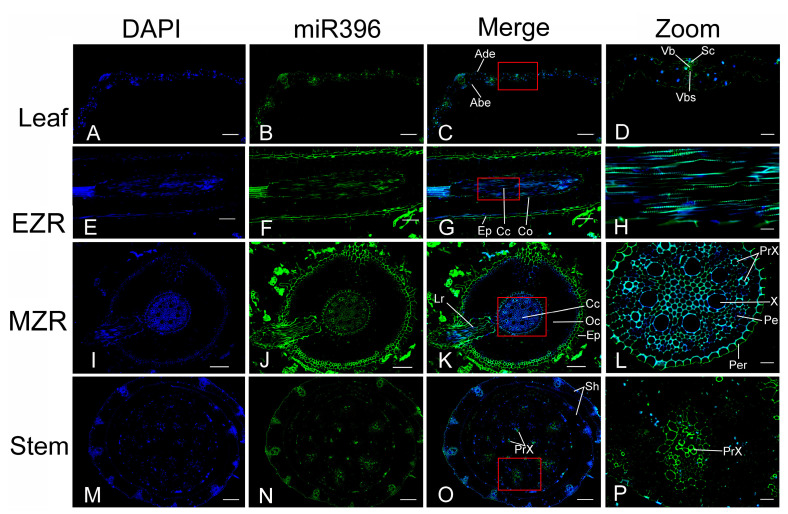
Verification of miR396 location in leaf, root, and internode tissues of the seedlings using FISH. Ped-miR396a-3p was expressed in the vascular tissues (**A**–**D**) of the leaves, the elongation (**E**–**H**) and mature zones (**I**–**L**) of the root tips, and the stems (**M**–**P**). Abe: abaxial epidermis; Ade: adaxial epidermis; Cc: central cylinder; Co: cortex; EZP: elongation zone of root; Ep: epidermis; Lr: lateral root; MZR: mature zone of root; Pe: phloem; Per: pericycle; PrX: protoxylem; Sc: sclerenchyma; Vb: vascular bundle; Vbs: vascular. Scale bar in panels (**A**–**C**,**E**–**K**,**M**–**O**) is 100 µm, and in panels (**D**,**H**,**L**,**P**) is 20 µm.

After excluding the background signals from the controls, different fluorescence intensities in the merged images were noticed in different tissues of the internodes, leaves, and roots of the seedlings (Figure S1). The fluorescence intensity of ped-miR396d-5p was higher than that of 4,6-diamidino-2-phenylindole 2 HCl (DAPI) staining mainly in the cell wall of the lower epidermal, upper epidermal, and vascular tissues in the leaf (Figure 6A–D). In the root, the signal was particularly strong in the walls of the epidermal, cortex, and vascular tissues in the elongation and maturation zones and in the lateral roots produced by the pericycle in the maturation zone (Figure 6E–L). In the stem, strong signals were found in annular thickening rings of the protoxylem vessels and the tracheids of the stem and the xylems of the sheath (Figure 6M–P) of the seedlings. These findings indicated that ped-miR396d-5p was expressed specifically in the stem, root, and leaf vascular tissues of the seedlings.

## 3. Discussion

This study assessed the expression patterns of bamboo miR396s, projected their target genes, and delineated the miR396-mRNA network in tissue differentiation of Moso bamboo.

### 3.1. Bamboo miR396-mRNA Regulatory Module in Vascular Tissues

GRFs are transcription factors specific to plants. In *Arabidopsis* and rice, the GRF levels can be regulated by miR396s. High miR396 levels affect cell elongation and proliferation due to the quantitative effects caused by GRF depletion in the *Arabidopsis* roots [28] and leaves [29,30]. Kim et al. [27] suggested that miR396 dramatically downregulated *GRF* expression during leaf development and reduced leaf blade length under elevated CO_2_. Despite these findings, the effects of the miR396-GRF regulatory module remain largely unknown.

The present study found that the GRFs were engaged with most miR396-mRNA pairs in the regulatory network. Moreover, miR396 cleavage sites were identified in five putative GRF genes via degradome sequencing. All these genes contain a DNA-targeting WRC domain [31,32] and a QLQ domain, that recruits ATP-dependent DNA translocase Switch/Sucrose Non-fermenting (SWI/SNF) via interacting with GRF-interacting factor (GIF) [33,34], thereby remodeling chromatins. During primary thickening, stem cells in the shoot apical meristem produce rapidly dividing daughter cells. Possibly, *PeGRF* forms complexes with GIFs and SWI/SNF, which would ease the association between DNA and the histone octamers, facilitating the expression of the genes responsible for the stem cell transition to dividing daughter cells. Additionally, the shoot meristematic zone exhibited both cell proliferation and cell elongation; these combined effects determine the rate of tissue organization. Our previous results showed that gibberellin acid (GA) concentration varied significantly between stages in the thickening shoots. Lu et al. [35] found that the miR396-GRF-GIF-SWI/SNF module regulates GA signaling in rice while the DELLA protein, the GA signaling pivot, plays dual roles in controlling the GRF levels by regulating the miR396 level and interacting with the GRFs. These findings shed light on the involvement of the miRNA-GRF regulatory network in the primary thickening of bamboo shoots.

Notably, miR396s also cleave targets related to the cell wall organization and cell homeostasis. For example, ped-miR396a-5p can cleave *PH02Gene30999*, which encodes a putative bamboo feruloyl esterase (FAE) protein. FAEs catalyze the hydrolysis of plant cell wall polysaccharides and regulate the release of ferulic acid, a natural antioxidant from plant cell walls [36]. MiR396s may affect the expression of FAEs and participate in cell wall rigidity and strength. Additionally, ped-miR396e-5p can cleave PH02Gene40368, which encodes a putative gene involved in potassium ion transmembrane transport. In *Arabidopsis*, potassium deficiency evokes two successive yet distinct calcium signals in the post-meristematic stelar tissues and refines tissues in the elongation and root hair differentiation zones, exhibiting spatial and temporal specificity [37]. Potassium homeostasis in plants is strictly regulated against a concentration gradient in the environment. Indeed, tissue differentiation in the thickening bamboo shoots is accompanied by environmental changes. These observations indicate an essential and direct role of potassium homeostasis for tissue development and point to a complex role of miR396 in thickening bamboo shoot growth and homeostasis.

### 3.2. Potential Role of Bamboo miR396 in Vascular Tissue Differentiation

Bamboo plants differ from woody trees by lacking developed secondary cambia and having distinctive tall culms with luxuriant leaves. Unlike most other monocot herbs, they are characterized by a complex and vast system of underground rhizomes. Therefore, they have a strong and unimpeded vascular system throughout the whole plant to satisfy source-sink balance demands and knotty culms with multiple layers of vascular tissue to resist external mechanical stresses. Thus, the molecular regulation of vascular systems in bamboo plants is species-specific and more complex than that in other dicotyledonous plants.

MiRNAs play important roles during plant evolution in instituting plant cell identity and organ morphology [38]. Indeed, previous reports indicated that miR396s demonstrate regulatory effects in a species-specific manner. For example, *Arabidopsis* miR396 prevents oxidative damage caused by brassinosteroids during seedling de-etiolation [39]; Rice miR396 augments grain yield by shaping inflorescence architecture [40]; Medicago miR396 affects mycorrhizal associations and root meristem activity [41]; Agrostis miR396 participates in vernalization and flower development [42]. Until now, the role of the miR396-mRNA regulatory module in Moso bamboo has remained unclear.

The present study showed that most of the target genes of five miR396 members were significantly up- or down-regulated in pairwise combinations of S3 in the WTTW group. The TW Moso bamboo is a stable variety of WT Moso bamboo and features a thicker culm wall with more vascular bundles and a narrower cavity. Up to the S3 stage, the cross-sectional area ratio of the culm wall to the cavity continues to decrease [14]. RNA FISH localized miR396 in the vascular tissues of the root, leaf, and stem of bamboo seedlings. Li et al. [16] showed that most miR166s’ target genes were significantly up- or down-regulated in S2_vs_S1 for both the WT and TW groups. Interestingly, RNA FISH also localized miR166 in the vascular tissues of the underground thickening shoots of the two-year-old seedlings [43]. MiRNAs may work synergistically to regulate bamboo vascular differentiation in different stages. These findings emphasize the complexity of the molecular regulatory network for vascular differentiation in Moso bamboo.

MiRNA biogenesis is regulated by Rbfox proteins via sequence-specific binding to their precursors and the targeting of downstream Dicer [44]. Changes in the pre-miRNA secondary structures could affect cleavage accuracy, rate, or both [45]. The present study identified many mutations in the precursor sequences between rice and Moso bamboo, leading to changes in their secondary structures. Additionally, more sophisticated molecular regulatory networks and mechanisms may have evolved in the miRNA evolution in Moso bamboo since the rhizome buds reproduce mainly asexually. The cleavage accuracy, rate, or both may have differed between *B. distachyon* and rice, two evolutionarily closely related species, resulting in extremely different morphological outcomes. Overall, our results emphasize the role of miR396 in primary thickening shoots and provide insights into the morphological diversity in bamboo.

### 3.3. Potential Role of miR396 in Xylem Patterning

Previous studies have indicated that miRNAs function as mobile signals and determine the fate of the xylem cells during development. In *Arabidopsis*, although miR165/6 is produced in root endodermis, its gradient expression toward the vascular center results in an opposite gradient expression of its target genes [46]. A previous study by Mi et al. [47] indicated that AGO1 preferentially recruits small RNAs with 5’ uridine to form the silencing complexes and control the gene expressions at both the transcriptional and post-transcriptional levels. Fan et al. [48] examined the loading of mobile miRNAs on cytoplasmic AGO1 and showed that miR165/6 migrated into the vasculature, affecting the fate of the xylem cells in the roots of *Arabidopsis*. Our study identified three overexpressed miR396s with the first base being uridine at the 5′ terminal. RNA FISH located ped-miR396d-5p in the root endodermis and xylem, the stem protoxylem vessels, and the leaf vascular tissues of bamboo seedlings. Thus, recruiting miR396s by bamboo AGO1 to constitute miRISCs could affect a series of target genes at the expression level and function as a local and long-distance signal-regulating multiple organ patterning in bamboo xylem.

Notably, the primary underground thickening bamboo shoots developed from rhizome buds to resemble the morphology of the aboveground culm. We detected ped-miR166a-3p, which holds a 5′ uridine at the first position, in the procambium and vascular tissues in the stem endodermis and shoot xylem at the early stages and in the central cylinder of the root tips from seedlings [16]. Bamboo miR396 members with a 5′ uridine at the first base could behave like *Arabidopsis* miR166/5; they might move freely through the bamboo xylem to be transported to other vasculatures and function as the mobile and morphogenic signals, together with miR166/5, determining the fate of the cells during development. Detailed analysis and further experiments are needed to investigate the role of miR396 and its target genes in xylem patterning.

## 4. Materials and Methods

### 4.1. Plant Materials

Moso bamboo plants were grown in a conditioned greenhouse in the National State Forestry and Grassland Administration Key Open Laboratory at the Science and Technology of Bamboo and Rattan, Beijing, China (N: 39°59′17.52″, E: 116°28′46.06″) at 25 ± 1 °C with 60 ± 5% relative humidity and 16:8 h light:dark cycles under 1350–1600 µmol m^−2^ s^−1^ illumination and irrigated three times per week. At two months of growth, the stems, roots, and leaves were collected and used to explore the localization of miR396s.

### 4.2. Data Collection

The sequencing data of small RNAs, transcriptome, and degradome of the underground thickening shoots of WT and TW Moso bamboo at five different developmental stages were downloaded from the NCBI SRA database (https://www.ncbi.nlm.nih.gov/Traces/study/?acc=PRJNA753616; accessed on 6 October 2022) Li et al. [16].

### 4.3. MiR396 Identification and Annotation

The downloaded sequencing reads of small RNAs were blasted against RNA databases, including Rfam (http://rfam.xfam.org/; accessed on 6 October 2022), Silva (http://www.arb-silva.de/; accessed on 6 October 2022), GtRNAdb (http://lowelab.ucsc.edu/GtRNAdb/; accessed on 6 October 2022), and Repbase (http://www.girinst.org/repbase/; accessed on 6 October 2022) using Bowtie v1.0.0 [49] with parameters’-v 0’ to identify tRNAs, rRNAs, snoRNAs, snRNAs, other ncRNAs, and repeat sequences. Because bamboo miRNAs have not been deposited in miRbase and annotated in the Moso bamboo genome, to identify well-recognized miRNAs, unannotated reads were mapped onto the Moso bamboo reference genome v2.0 [50]. The mapped miRNAs were further aligned using Bowtie v1.0.0 to miRNAs in miRbase [51] (release 22, http://mirbase.org/; accessed on 6 October 2022) from *Brachypodium distachyon* and *Oryza sativa*, which are most closely related evolutionarily to Moso bamboo.

To predict novel miRNAs, unaligned unique reads were analyzed using miRDeep-P2 [52] as described previously [53] based on the updated criteria for plant miRNAs [54]. MiRNAs of similar sequences were clustered into the same family.

### 4.4. Analysis of miR396 Expression

MiR396 expression was normalized based on TPM algorithm [55] and calculated using the formula below:

TPM = Readcount × 1,000,000/Mapped Reads(1)

where Readcount is the number of miRNAs in the reference genome v2.0, and Mapped Reads are the number of reads mapped to a particular miRNA.

Replicates with high consistency (*r*^2^ > 0.70) between each other were further analyzed based on Pearson’s correlation analysis for miRNA expression.

Expression levels of miRNAs between different combinations were compared using DESeq2 [56]. After filtering out miRNAs with low expression, miRNAs with TPM > 1 in either one of the combinations, |log2(foldchange)| ≥ 1.00, and a false discovery rate (FDR) corrected *p*-value < 0.05 were considered differentially expressed miRNAs (DEmiRs) and presented as “X_vs_Y”. For example, X01_vs_X02 means the DEmiRs between X01 and X02. A total of 13 combinations were analyzed, including WTS2_vs_WTS1, WTS3_vs_WTS2, WTS4_vs_WTS3, WTS5_vs_WTS4, TWS2_vs_TWS1, TWS3_vs_TWS2, TWS4_vs_TWS3, TWS5_vs_TWS4, TWS1_vs_WTS1, TWS2_vs_WTS2, TWS3_vs_WTS3, TWS4_vs_WTS4, and TWS5_vs_WTS5.

### 4.5. Target Prediction and Annotation

To identify potential targets for miR396, psRNATarget [57] with the default parameters of Schema V2 (2017 release) was used except when the threshold value of 3 was used for a more restricted expectation.

For annotation, the potential targets were queried against various databases, including Pfam (http://pfam.xfam.org/; accessed on 10 October 2022), GO (http://www.geneontology.org/; accessed on 10 October 2022), NR (ftp://ftp.ncbi.nih.gov/blast/db/; accessed on 10 October 2022), and KEGG (http://www.genome.jp/kegg/; accessed on 10 October 2022) using BLAST v2.2.26 [58].

### 4.6. Expression Analysis of the Predicted Targets

HISAT2 v2.0.4 (Kim et al., 2015) was used to map the reads downloaded from the transcriptome sequencing database to the Moso bamboo reference genome v2.0 with parameters ‘--dta -p 6 --max-intronlen 5000000’ for known gene identification. Reads in the unannotated regions were assembled using StringTie v1.3.4d [59] for novel gene identification with parameters ‘--merge -F 0.1 -T 0.1’.

Fragments Per Kilobase of transcript per Million fragments mapped (FPKM) (Florea et al., 2013) was used to estimate gene expression levels based on Formula (2):

FPKM = cDNA Fragments/Maped Fragments (Millions) × Transcript Length (kb)(2)

Replicate reads with high consistency (*r*^2^ > 0.70) between each other were further analyzed based on Pearson’s correlation analysis for gene expression.

Differentially expressed genes (DEGs) were defined as genes expressed (FPKM > 1) in either of two pairwise combinations with |log2(FC)| ≥ 1.00 and an FDR corrected *p*-value < 0.01 and identified using DESeq2 [56] after removing low expression genes.

### 4.7. Degradome Analysis of miR396-mRNA Interaction Pairs

The reads downloaded from the degradome sequencing database were aligned using Bowtie v1.0.0 to non-coding RNA sequences from Rfam (except miRNA). After removing the reads that were aligned to repeats, tRNAs, rRNAs, and snoRNAs, the remaining reads were further aligned against the v2.0 Moso bamboo reference genome with maximum one mismatch and subjected to analysis using the CleaveLand (v4.4) pipeline [60] to identify potential miR396 cleavage sites. Only results from categories 0, 1, and 2 were considered to maximally eliminate false positives.

### 4.8. Sequence Analysis and Phylogenetic Tree Construction

Gene structures were analyzed based on the annotation files downloaded from bambooGDB (http://www.bamboogdb.org; accessed on 16 February 2022). Protein domains were projected using SMART (http://smart.embl-heidelberg.de; accessed on 11 October 2022). Phylogenetic trees were constructed using MEGA v5 [61] using the neighbor-joining (NJ) method with 1000 bootstrap replicates.

### 4.9. Dual-Luciferase Reporter Assays in N. benthamiana Leaves

Total RNA was isolated using Tiangen RNAprep plant kit (Tiangen, Beijing, China) from underground shoots collected from two-year-old bamboo plants and treated with RNase-free DNase I (Tiangen). cDNA was converted from 1 μg RNA using TransScript^®^ II One-Step gDNA Removal and cDNA Synthesis SuperMix (TransGen Biotech).

Luciferase reporter assays were conducted as reported [62] with slight modifications, including vector, restriction enzyme and plasmid. *PeGRF6* sequence with the predicted target site was inserted into the pGreenII 0800-miRNA vector [63] between *Kpn*I and *Eco*RI. The precursor sequence of ped-miR396d-5p was inserted behind the 35S promoter of the pCAMBIA1300 vector. pGreenII-0800::PeGRF6 and 35S::ped-miR396d-5p vectors were transformed into *Agrobacteria* GV3101 (pSoup) and GV3101, respectively. The transformed strains were co-infiltrated into the leaves of *N. benthamiana*. Empty pCAMBIA1300 vectors were used as the controls. At 48 h after infiltration, fluorescent signals were detected and compared. Table S5 shows the primers.

### 4.10. RNA FISH

To observe the location of miR396s, various seedling tissues were subjected to FISH assays using the FISH kit (C007, Shanghai Gefan Biotechnology Co., Ltd., Shanghai, China). In brief, paraffin-embedded sections were prepared, as previously reported by Wei et al. [14]. Before hybridization, the sections were dewaxed, incubated for 10 min at 22 °C in a solution of 30% (*w*/*v*) H_2_O_2_ and methanol (1:10, *v*/*v*), treated with proteinase K for 20 min at 37 °C, fixed in 4% (*w*/*v*) paraformaldehyde, and washed with PBS. After being treated with acetic anhydride, the sections were incubated overnight at 65 °C with a FITC-labeled probe (5′-CAGUUCAAGAAAGCUGUGGAA-3′, Abiocenter, Beijing, China). After washing with 2 × SSC buffer, the sections were further washed with formamide/4 × SSC (1:1, *v*/*v*) and PBS and subjected to counterstaining for nuclei with DAPI (Invitrogen). After washing with PBS, the stained sections were mounted on the slides using antifade buffer (Beyotime, Shanghai, China), observed under a Nikon Eclipse Ci-L microscope (Tokyo, Japan), and photographed.

### 4.11. Statistical Analyses

Data were processed using SPSS 19.0 software (IBM, Armonk, NY, USA) and presented as means ± standard deviation (SD). Independent samples *t*-test was used to compare the means of two samples. Differences in transcript and small RNA sequencing data were compared with single factor analysis of variance. The raw reads obtained from the RNA-seq data were subjected to differential expression analysis using DESeq2 software [56]. For simulating read counts, the DESeq2 software uses negative binomial distribution [64]. Differences with FDR [65,66] corrected *p*-value < 0.05 between groups were regarded as statistical significance.

## 5. Conclusions

We assessed the changes in the expression levels, profiles, and potential targets of miR396s and elucidated the miR396-mRNA network in thickening bamboo shoots. Our results suggest a critical role of miR396s in the signalling pathways during bamboo tissue differentiation. Further, the xylem-specific location of ped-miR396a-3p in the stems and roots emphasized their roles in bamboo xylem development. However, the interactions between miR396s and their predicated targets should be further confirmed experimentally, and genetic transformation is required to explore the role of miR396 and its predicted targets. In conclusion, based on our results, we propose miR396s and *PeGRFs* as key candidates for the genetic improvement of bamboo wood properties and edible quality. Our present findings revealed a complex network in which miR396s regulated *PeGRFs*, *PePHDs*, and other genes involved in the signalling pathways, therefore controlling tissue differentiation in bamboo.

## Data Availability

The nucleotide sequence of *PH02Gene06203* encoding a putative *PeGRF6* transcription factor is available in GenBank with the accession number OQ026341.

## Supplementary Materials

The following supporting information can be downloaded at: https://www.mdpi.com/article/10.3390/plants12051103/s1, Supplemental Figure S1: Signals of blank control samples in fluorescence in situ hybridization (FISH) analysis; Supplemental Table S1: Five miR396s that were identified by sequencing the small RNAs isolated from the underground thickening wild-type (WT) and thick wall (TW) variant shoots. Three miR396s that were overexpressed (TPM > 500) at all stages are bolded. NA: not available. a: average of the data. U: up-regulated; D: down-regulated; Supplemental Table S2: The 1285 predicted targets of seven miR396s. Bold, red, and italics letters indicate TF, PK, and TR targets, respectively; Supplemental Table S3: 115 pairs of differentially expressed miR396s (DEmiRs) and genes (DEGs) were identified. Among them, 57 miRNA-mRNA coherently expressed pairs are shown in bold. Two overexpressed miR396s with TPM > 1000 at all stages are shown in red. Bold, red, and italic letters indicate TF, PK, and TR targets, respectively. a: average of the data. U: up-regulated; D: down-regulated; Supplemental Table S4: Ten miR396-mRNA pairs were verified in degradome sequencing dataset; Table S5: Primer sequences used for the dual-luciferase reporter assay.
