# Supplementary figures and images for: Network of miR396-mRNA in Tissue Differentiation in Moso Bamboo (Phyllostachys edulis)
Source: Plants (Basel). 2023 Mar 1;12(5):1103. doi: 10.3390/plants12051103 (PMC10005394; doi:10.3390/plants12051103)

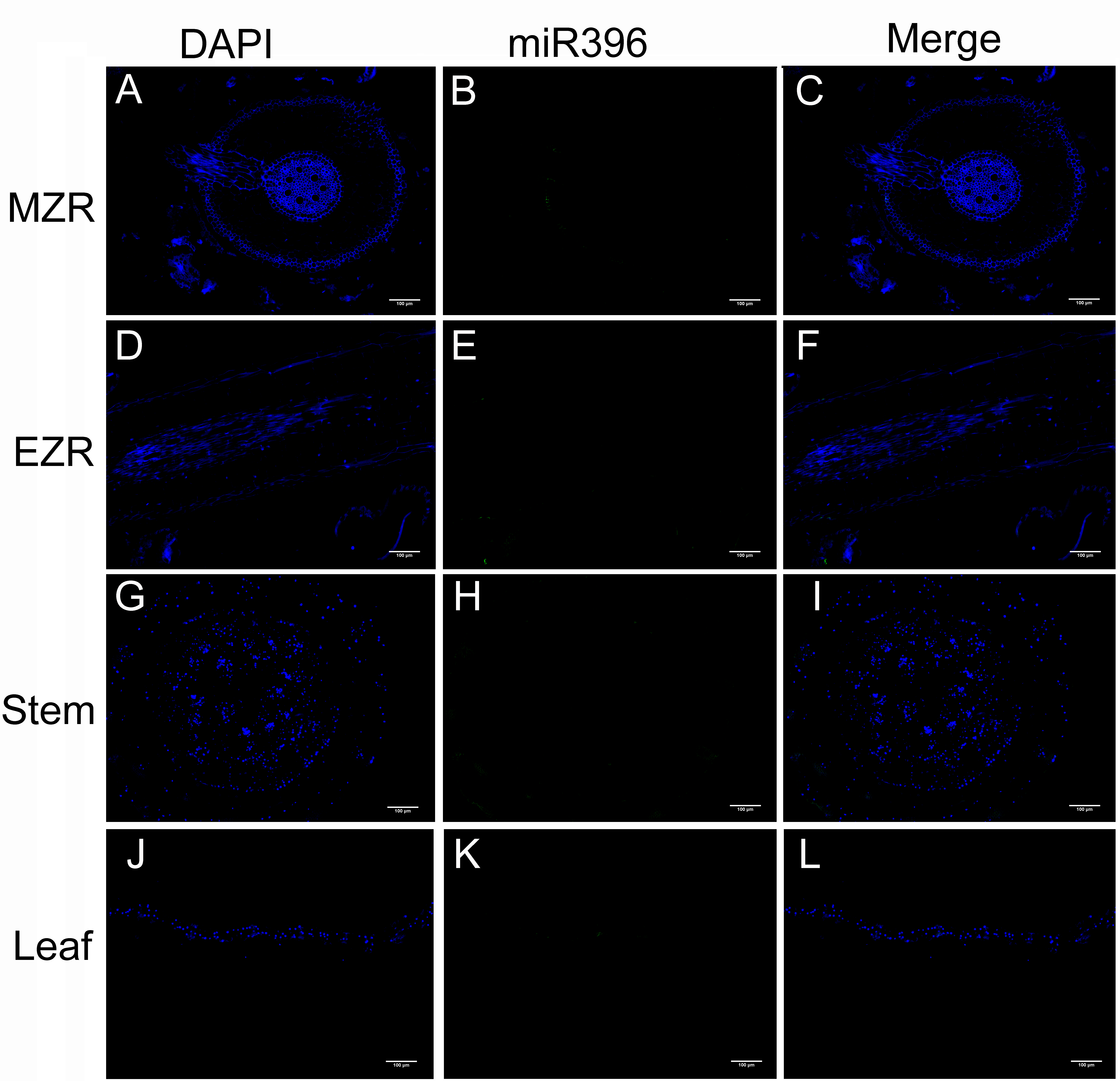

Supplement: Supplementary file 1 [file plants-12-01103-s001.zip › Figure S1.tif]
